# Effects of suspended sediments on the sponge holobiont with implications for dredging management

**DOI:** 10.1038/s41598-017-05241-z

**Published:** 2017-07-10

**Authors:** Mari-Carmen Pineda, Brian Strehlow, Miriam Sternel, Alan Duckworth, Ross Jones, Nicole S. Webster

**Affiliations:** 1 Australian Institute of Marine Science (AIMS), Townsville, QLD and Perth, WA Australia; 2 Western Australian Marine Science Institution, Perth, WA Australia; 3 Centre for Microscopy Characterisation and Analysis, School of Plant Biology and Oceans Institute, University of Western Australia, Crawley, WA Australia; 4 University of Bremen, Bremen, Germany

## Abstract

Dredging can cause high suspended sediment concentrations (SSC) in the water column, posing a hazard to filter feeding organisms like sponges as sediment may clog their aquiferous systems and reduce feeding. In order to provide pressure−response values for sponges to SSC and tease apart the cause:effect pathways of dredging pressures, five heterotrophic and phototrophic species were experimentally exposed to a range of dredging-relevant SSC of up to 100 mg L^−1^, with light compensation across treatments to ensure that SSC was the primary physical parameter. This study shows that some sponge species exposed to high SSC (≥23 mg L^−1^) for extended periods (28 d) have lower survival, increased necrosis and depletion of energy reserves. In contrast, SSC of ≤10 mg L^−1^ caused few, if any, negative effects and is thus suggested as a prudent sub-lethal threshold for sponges. Microbial communities did not change significantly among SSC treatments, although a nutritional shift from mixotrophy towards increased phototrophy was detected for some sponge species exposed to high SSC. Importantly however, it is expected that the combined effect of SSC with low light availability and sediment smothering as occurs during dredging operations will increase the negative effects on sponges.

## Introduction

Dredging is an essential part of all port operations and the need for dredging is likely to increase with the current trend towards larger ships with deeper draft requirements^1^. World population growth and the related energy demands have also resulted in a new market for dredging associated with expanding port infrastructure to exploit remotely located fossil fuels deposits (CEDA 2013). In recent years there has been a very high demand for this type of dredging in tropical NW Australia associated with a resources boom and a burgeoning liquefied natural gas (LNG) industry^2, 3^.

Capital and maintenance dredging, and a suite of dredging-related activities such as bed levelling, trench digging for gas pipe lines, and especially dredge material placement, releases sediment into the water column. The cloudy (turbid) plumes that are generated can drift onto nearby benthic habitats, posing risks to ecologically important marine ecosystems such as coral reefs, seagrass meadows and sponge gardens^4–8^. The effect of suspended sediment on sponges in NW Australia is particularly interesting, because macrobenthic filter feeders can dominate in many locations^9, 10^. The response of sponges to the increased turbidity is not well known^11^, and this is a significant challenge to their effective management^12^.

Sponges are sessile filter-feeding organisms playing important roles in many marine ecosystems, including substrate consolidation, habitat provision, seawater filtration, and bentho-pelagic energy transfer^11, 13^. They obtain energy heterotrophically by filtering seawater through an aquiferous system or internal canal network^14, 15^. Many sponges also obtain energy autotrophically from photosymbionts, of which *Cyanobacteria* are the most ubiquitous^16–20^, encompassing sponge-specific groups such as ‘Candidatus *Synechococcus spongiarum’* and *Oscillatoria spongeliae* ^21–24^. Dinoflagellates of the genus *Symbiodinium* are also known to be photosymbionts of sponges, especially in bioeroding sponge species^19, 25, 26^. In some tropical sponge species the photosymbionts can provide >50% of the energy requirements of the host^18, 27^, and the diverse symbiotic consortia can comprise up to 40% of the sponge volume^28^. Sponge symbionts tend to be highly host specific, but are generally stable across broad geographic and environmental gradients^29^, with the stable host-microbe consortium often referred to as the ‘sponge holobiont’^28^.

Depending on their degree of nutritional dependence from symbiont primary production, the sponge holobiont can be described as either ‘phototrophic’, ‘mixotrophic’ or ‘heterotrophic’. Some species exhibit considerable flexibility in their feeding strategy and are able to alter their nutritional mode depending on prevailing conditions^27, 30, 31^. The different modes of nutrition have important implications for understanding the environmental effects of dredging activities on sponges. Due to their filter feeding activity, sponges may be affected by elevated suspended sediment concentrations (SSCs) and long term exposure to high SSCs can lead to clogging of their aquiferous systems, reducing the flow of oxygenated seawater to the sponge mesohyl and compromising heterotrophic feeding^32^. Some sponges exhibit short term responses to high turbidity such as temporarily closing or reducing the size of their incurrent openings (ostia) and arresting pumping (filtering) activity^33–36^. However, reduced pumping activity has flow-on consequences for host energetics, health and reproductive output^32, 37–39^. Since some sponges also rely on photoautrotrophy for nutrition, they may be affected by the pronounced reduction in benthic light availability that occurs in dredging plumes^4, 40, 41^. Periods of low light levels associated with dredging can cause photoacclimation of the photosymbionts^32, 42, 43^, but extended periods of low light can also result in dissociation of the holobiont (bleaching) and subsequent mortality in some phototrophic species^44^.

Understanding the effects of turbidity on sponges would facilitate development of water quality thresholds (or trigger values) that could be used to alert dredging proponents of conditions that could, if continued, cause environmental harm. Depending upon permit requirements, the option could then be to modify the dredging operations i.e. changing the rate or location of dredging, and hence avoid or minimize impacts. Developing thresholds is predicated upon identifying key cause-effect pathways and determination of dose−response relationships which is technically challenging. Close to dredging activities, sponges will be exposed to both high SSCs in combination with low light conditions (caused by the water turbidity), and may also be simultaneously exposed to high levels of sedimentation and possibly smothering. There are, therefore, many potential cause-effect pathways. In order to avoid conflation of these in a single experiment (see ref. 4), in this study we examine the response of sponges to a range of suspended sediments but kept light uniform across treatments. Experiments were conducted over 28 d, with a similar suite of 3 heterotrophic and 2 phototrophic sponges as used in previous studies assessing the effects of sedimentation^38^ and light^44^.

## Results

### Physical parameters

**Figure 1:**
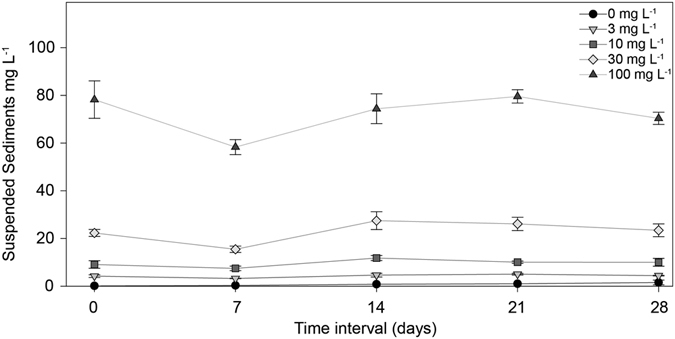
SSC during the experiment. (**a**) Means ( ± SE) SSCs (mg L^−1^) at each treatment, according to gravimetric measures.

The nominal SSCs (0, 3, 10, 30 and 100 mg L^−1^) were largely constant over time and varied significantly among treatments (ANOVA: *P* < 0.001; Fig. 1). Gravimetric analysis of SSCs indicated the tanks containing sediment (0.4 ± 0.04 mg L^−1^, 4.3 ± 0.2 mg L^−1^, 9.7 ± 0.6 mg L^−1^, 23 ± 1.2 mg L^−1^, 73 ± 2.8 mg L^−1^, mean ± SE) were generally slightly lower than the desired concentrations, especially for the two highest treatments (Fig. 1). For simplicity, we refer to the nominal SSCs throughout the manuscript.

### Sponge growth, necrosis and mortality

**Figure 2:**
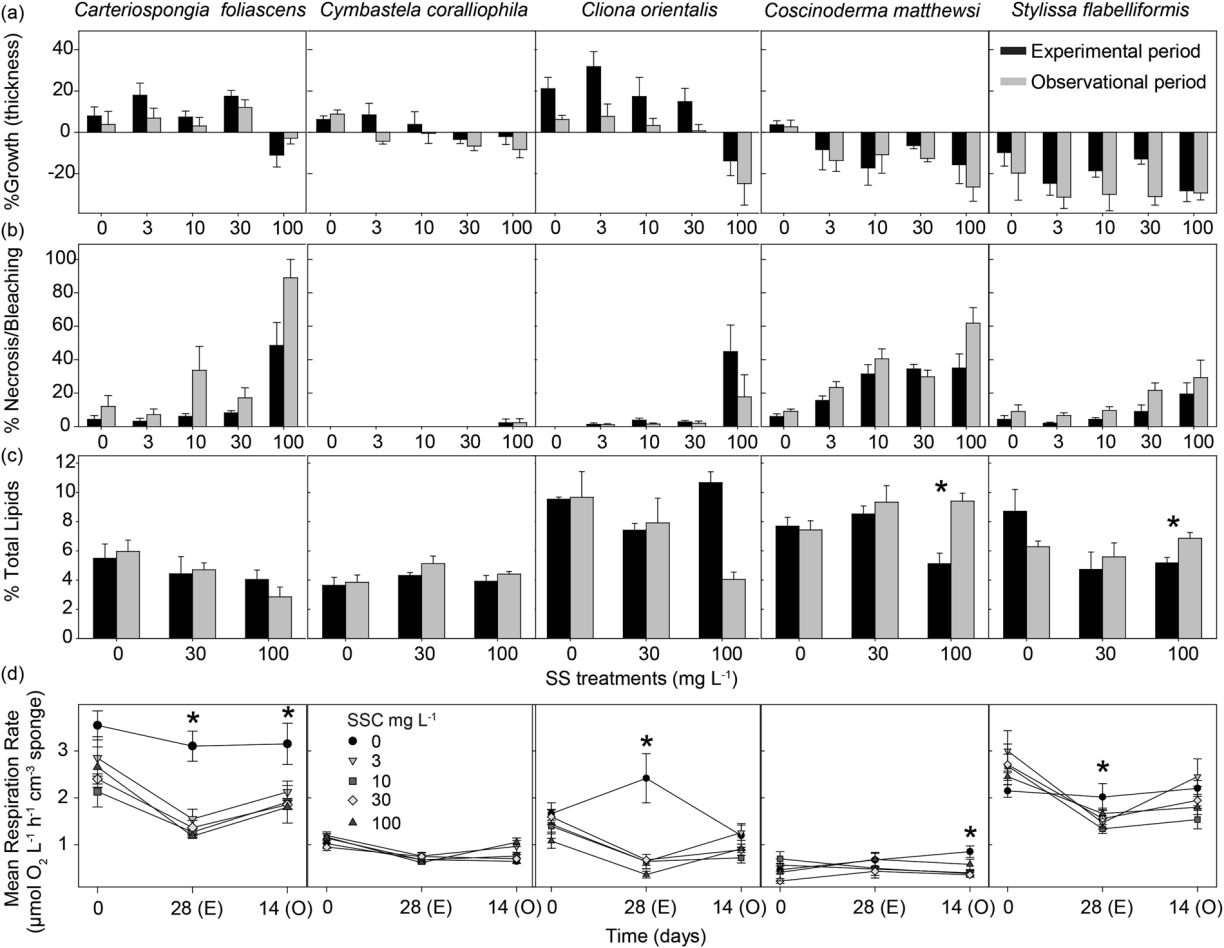
Physiological responses of sponges to elevated SSCs. (**a**) Percentage of growth (mean ± SE) (based on sponge tissue thickness), (**b**) Percentage of necrotic or bleached tissue, (**c**) Percentage of sponge biomass comprised of lipids, and (**d**) Mean respiration rates (µmol O_2_ L^−1^ h^−1^ cm^−3^ sponge), for all species and SSCs (0, 3, 10, 30 and 100 mg L^−1^) after the 28 d experimental period (**E**), and 14 d observational period (O) (mean ± SE). Asterisks show statistically significant differences between the experimental and observational phase in a–c and between treatments in d (t-tests: *P* < 0.05).

Most sponges appeared visibly healthy at the end of the experiment, with the exception of all *Carteriospongia foliascens*, a few individuals of *Coscinoderma matthewsi*, and *Cliona orientalis* exposed to 100 mg L^−1^. No visible effects were observed in *Cymbastela coralliophila* and *Stylissa flabelliformis* in any treatment. Percentage growth and relative growth rates based on thickness measurements before and after the 28 d sediment exposure and observational period, were negative for most sponges exposed to SSC of ≥30 mg L^−1^; however with the exception of *Cliona orientalis*, these negative growth rates were not significantly different from control groups (Fig. 2a, Table 1a). The bioeroding sponge *Cliona orientalis* grew during the sediment exposure and recovery periods in all treatments except at the highest SSC of 100 mg L^−1^ (Fig. 2a, Table 1a). The massive sponge *Coscinoderma matthewsi* shrank when exposed to suspended sediments (3–100 mg L^−1^) and only grew in the control treatment, although this growth was not statistically significant. The erect sponge *Stylissa flabelliformis* shrank in all treatments with tissue regression increasing with increasing SSC and no growth evident during the observational period (Fig. 2a, Table 1a). Sponge growth based on surface area (SA) for all species showed similar results, generally shrinking in the higher SSC treatments (Supplementary Fig. S1, Table S1).

**Table 1:** ANOVA tables and summaries of linear mixed models testing the effects of different SSCs on the physiological responses of sponges.

Source	*df*	*Carteriospongia foliascens*		*Cymbastela coralliophila*		*Cliona orientalis*		*Coscinoderma matthewsi*		*Stylissa flabelliformis*	
		*F*	*P*	*F*	*P*	*F*	*P*	*F*	*P*	*F*	*P*
(A) Relative growth rate (thickness)											
Experimental phase											
Treatment	4	3.337	0.109	1.47	0.337	5.81	0.04	0.767	0.589	1.752	0.275
Error	20										
Tukey						0,3,10,30 > 100					
Observational phase											
Treatment	4	2.761	0.148	4.319	0.07	3.94	0.08	1.887	0.251	0.251	0.898
Error	20										
(B) Percentage of necrosis and bleaching											
Experimental phase											
Treatment	4	9.12	<0.0001	0.8	0.574	7.25	0.02	7.07	0.02	3.64	0.09
Error	20										
Tukey		0,3,10,30 < 100				0,3,10,30 < 100		0 < 3,10,30,100			
Observational phase											
Treatment	4	9.80	0.01	0.8	0.573	1.598	0.306	12.9	<0.0001	3.14	0.12
Error	20										
Tukey		0,3,10,30 < 100						0,3 < 10,100			
(C) Percentage of sponge biomass comprised of lipids											
Experimental phase											
Treatment	2	0.612	0.598	0.733	0.550	10.95	0.04	4.837	0.115	3.783	0.151
Error	12										
Tukey						0,100 > 30					
Observational phase											
Treatment	2	5.255	0.105	2.220	0.256	7.212	0.071	1.951	0.287	0.998	0.465
Error	12										
(D) Respiration Rates											
Treatment	4	6.920	0.0285	1.832	0.2605	2.076	0.2218	1.344	0.3701	0.7203	0.613
Time	2	17.177	<0.001	63.407	<0.001	16.51	<0.001	1.477	0.2406	38.153	<0.001
Treatm. x Time	8	0.6266	0.7494	3.581	0.00323	8.490	<0.001	4.118	0.0012	3.165	0.007
Error	40										
Tukey											
Treatment		0 > 10,30									
Time				T0 > E; T0 > O		T0 > E < O					
Treatm. within E						0 > 3,10,30,100					
Time within 0				T0 > E,O		T0 < E > O		T0 < O			
Time within 3				T0 > O > E		T0 > E,O				T0 > E < O	
Time within 10				T0 > E,O		T0 > E,O		T0 > O		T0 > E,O	
Time within 30				T0 > E,O		T0 > E, O				T0 > E,O	
Time within 100				T0 > E < O		T0 > E,O		T0 < E		T0 > E,O	

(A) Growth rate based on thickness measures, (B) Percentage of necrotic and bleached tissue, (C) Percentage of sponge biomass comprised of lipids, (D) Mean respiration rates, for each species separately, at the end of experimental phase (E) and observational phase (O). Tukey tests were performed for significant pairwise multiple comparisons. (SSC: 0, 3, 10, 30 and 100 mg L^−1^).

The percentage of dead (i.e. partial mortality or necrosis) and bleached (discoloured) tissue also increased significantly with higher SSC in all species except for *Cymbastela coralliophila* and *Stylissa flabelliformis* (Fig. 2b, Table 1b). The highest percentages of necrosed tissue were observed among individuals of *Carteriospongia foliascens* and *Coscinoderma matthewsi* exposed to the highest SSC, reaching 80 and 60% of sponge tissue respectively by the end of the observational period. *Carteriospongia foliascens* showed signs of necrosis from the second week of sediment exposure to 100 mg L^−1^, although some necrosis was also observed in lower treatments by the end of the experiment (Fig. 2b). In terms of total mortality, 90% of *Carteriospongia foliascens* died in the 100 mg L^−1^ treatment by the end of the observational period and 20% had died in each of the 30 and 10 mg L^−1^ treatments (Supplementary Fig. S2). The bioeroding sponge *Cliona orientalis* also showed signs of stress, as evidenced by significant bleaching, within the 100 mg L^−1^ treatment, although significant recovery of pigmentation occurred during the observational phase (ANOVA: *P* < 0.05). *Coscinoderma matthewsi* also exhibited considerable necrosis at high SSC including 20% total mortality in the 100 mg L^−1^ treatment by the end of the observational period (Supplementary Fig. S2). No mortality occurred in *Cymbastela coralliophila*, *Cliona orientalis* and *Stylissa flabelliformis*.

Mortality data for *Carteriospongia foliascens* was fitted to nonlinear regression curves to calculate the lethal concentration (LC) at which 50% (LC_50_) and 10% (LC_10_) of the population died. Nonlinear regression of the dose−response curve (R^2^ = 0.8675, AICc = 85.23) met assumptions of normality and homoscedasticity and there was no evidence for a lack of fit in the replicates test (*P* = 0.694). After the 28 d exposure period, the LC_50_ (and 95% confidence intervals range) for mortality in *Carteriospongia foliascens* was 40.6 mg L^−1^ (range: 28.9–57.0 mg L^−1^) and the LC_10_ was 21.5 mg L^−1^ (range: 13.1–35.2 mg L^−1^). Mortality data from *Coscinoderma matthewsi* did not meet model assumptions and LC values could not be calculated.

### Lipids

For all species, total lipid content was similar between time of sample collection and the start of the sediment dosing, as well as during the experiment for sponges exposed to no suspended sediment (ANOVA: *P* > 0.05). With the exception of *Cymbastela coralliophila*, all species had lower lipid content when exposed to SSCs ≥ 30 mg L^−1^, although this was only statistically significant for *Cliona orientalis* at the end of the 28 d sediment exposure (Fig. 2c, Table 1c). Unexpectedly, *Cliona orientalis* had a higher lipid content in the 100 mg L^−1^ treatment than in the 30 mg L^−1^ treatment and while lipid content appeared to decrease in samples from the 100 mg L^−1^ treatment during the observational phase, this decrease was not significant. *Coscinoderma matthewsi* and *Stylissa flabelliformis* increased their lipid content during the observational period (Fig. 2c).

### Respiration rates

Respiration rates did not significantly differ among treatments at day = 0 for any species (Fig. 2d, Table 1d). However, after 28 days, respiration rates were significantly lower for *Carteriospongia foliascens* and *Cliona orientalis* exposed to suspended sediment than conspecifics in the 0 mg L^−1^ treatment (ANOVA, *P* < 0.05; Fig. 2d). In *Carteriospongia foliascens*, respiration rates were lower than controls in the 10 and 30 mg L^−1^ treatments and the absence of a significant difference at 100 mg L^−1^ is likely due to the high mortality of *Carteriospongia foliascens* in this treatment (which precluded *post hoc* analysis, Table 1d). No interactive effect of time and treatment was observed for *Carteriospongia foliascens*. Respiration in *Cliona orientalis* in the 0 mg L^−1^ treatment increased significantly between day 0 and the end of the experimental phase, returning to pre-treatment levels in the observational period. Although this response in control sponges was unexpected, respiration rates were still observed to decrease for *Cliona orientalis* in all sediment treatments over time. Furthermore, respiration rates were significantly lower at the end of the observational phase than at day 0 within each sediment treatment, indicating that a longer recovery period was required for *Cliona orientalis* to return to pre-exposure respiration rates (Fig. 2d, Table 1d). A similar recovery capacity was observed in *Cymbastela coralliophila*, although increased respiration during the observational phase was only detected in the 3 mg L^−1^ treatment. Respiration rates in the massive species *Coscinoderma matthewsi* were significantly increased in sponges in the 100 mg L^−1^ treatment. *Coscinoderma matthewsi* was the only species to exhibit an increased respiration rate on exposure to SSC. Finally, respiration rates in *Stylissa flabelliformis* were not significantly different among treatments, although when looking within each sampling time individually, a significant decrease at the end of the 28 d was detected for all treatments exposed to sediments, with recovery only evident in the sponges exposed to the 3 mg L^−1^ treatment (Fig. 2d, Table 2d).

**Table 2:** ANOVA tables and summaries of linear mixed models on the effects of elevated SSCs on photosymbionts.

Source	*df*	*Carteriospongia foliascens*		*Cymbastela coralliophila*		*Cliona orientalis*	
		*F*	*P*	*F*	*P*	*F*	*P*
(A) Maximum quantum yield							
Treatment	4	1.238	0.4016	0.380	0.8154	2.912	0.1356
Time	6	3.410	0.0040	2.949	0.0101	2.523	0.0247
Treatm. × Time	18	1.468	0.0646	1.451	0.0985	2.582	<0.001
Error	96						
Tukey							
Time						Day 0 ≠ Day 14; O7 ≠ Day 7	
Time within 10 mg L-1						Day 0 ≠ Day 21	
Time within 100 mg L-1						Day 7 ≠ Day 0, 21, 28	
Treatment within Day 21(E)						0, 10 ≠ 100	
Treatment within Day 14(O)						0 ≠ 100	
(B) Chl a							
Experimental phase							
Treatment	4	5.400	0.0464	0.8582	0.5462	2.473	0.1738
Error	20						
Tukey		3, 10 > 100					
Observational phase							
Treatment	4	2.225	0.2018	2.238	0.2002	0.5241	0.7245
Error	20						
(C) PERMANOVA of all pigment data							
Treatment	4	4.738	0.001	1.698	0.078	2.383	0.021
Time(Treatment)	5	2.235	0.019	1.305	0.207	1.789	0.082
Error	40						
Pair-wise Tests		0 ≠ 3, 10, 30 ≠ 100				3 ≠ 10, 30, 100	

(A) Effects of treatment and time on maximum quantum yield throughout the experiment (B) Effects of treatment on Chl a concentrations at the end of the experimental (E) and observational (O) periods, and (C) Two-way PERMANOVA of all pigment data (Chl a, b, c, d, Total Chlorophyll and Carotenoids) with SSC and Time as factors, for the three phototrophic species (SSC: 0, 3, 10, 30 and 100 mg L^−1^). In a-c, Tukey tests were performed for significant pairwise multiple comparisons.

### Histology

Choanocyte chambers could only be accurately quantified in *Cliona orientalis*. While the number of choanocyte chambers at the end of the experimental period was 37.3 ± 11.6 and 9.7 ± 2.7, (mean ± SD) in sponges from the 0 mg L^−1^ and 100 mg L^−1^ treatments respectively, this difference was not statistically significant (t test: *P* = 0.103)(Supplementary Fig. S3).

### Chlorophyll fluorescence

**Figure 3:**
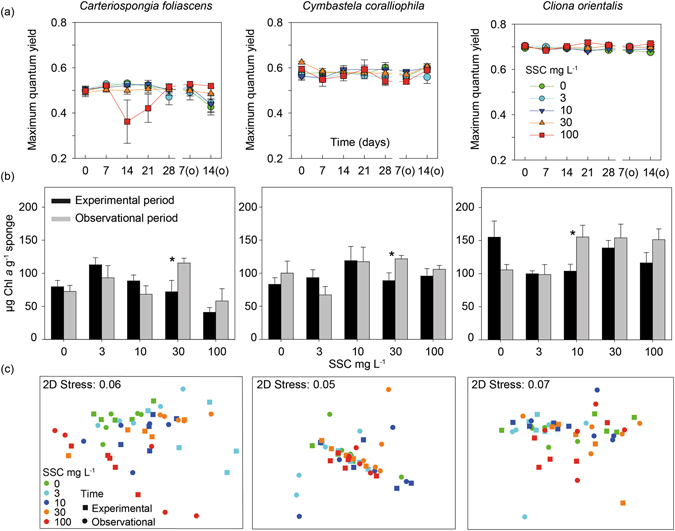
Response of the photosymbionts to elevated SSCs. (**a**) Mean values ( ± SE) of maximum quantum yield, (**b**) Mean values (±SE) of Chl *a*, and (**c**) Non-metric Multi-Dimensional Scaling (nMDS) of all photopigments retrieved by spectrophotometry, for the three phototrophic species and for all SSCs through the experimental phase and observational phase. Asterisks show statistically significant differences between the experimental and observational phase on b (t-tests: *P* < 0.05).

Overall, no significant differences in maximum quantum yield were observed between treatments for any of the phototrophic species hosting cyanobacterial symbionts, with minor fluctuations throughout the experiment and observational periods (Fig. 3a, Table 2a). For instance, lower quantum yields were measured from bleached *Carteriospongia foliascens* individuals on days 14 and 21, before they died, but while the decrease with time was significant, the interaction of time and treatment was not (Fig. 3a). In contrast, for *Cliona orientalis* which hosts *Symbiodinium* symbionts, the interaction term (treatment*time) was significant. Within the 10 mg L^−1^ treatment, quantum yields decreased significantly on day 14 and were also lower on observation day 7 than on day 7 of the exposure (Fig. 3a, Table 2a). Within the 100 mg L^−1^ treatment, quantum yields decreased after 7 days and then significantly increased after 21 days. After 21 days exposure, quantum yields in sponges from 0 and 10 mg L^−1^ treatments were significantly different from sponges in the 100 mg L^−1^ treatment. After 14 days of the observational period, quantum yields were significantly greater than in sponges exposed to 100 mg L^−1^ for 21 days.

### Pigment analysis

Chl *a*, was highly correlated with Total chlorophyll for *Carteriospongia foliascens*, *Cymbastela coralliophila* and *Cliona orientalis* (R^2^ > 0.85, *P* < 0.001), so Chl a concentration was used as a proxy for total photosymbiont health (i.e. bleaching). Overall, concentrations of Chl *a* were stable throughout the experiment (Fig. 3b). Significant differences in Chl *a* among SSC treatments were only observed for *Carteriospongia foliascens* at the end of the 28 d sediment exposure period, with lower concentrations in individuals exposed to 100 mg L^−1^ (Table 2b). However, a negative and significant correlation between Chl *a* and Chl *d* in both *Carteriospongia foliascens* and *Cymbastela coralliophila* (R^2^ = −0.254, −0.265, and *P* = 0.046, 0.037, respectively), and an increase in Chl *d* in samples exposed to 100 mg L^−1^ in both species (Supplementary Fig. S4), suggests an increase in some Chl *d*-containing Cyanobacteria under high SSCs. Chl *a* concentration in samples exposed to high SSCs returned to control levels following the observational period for all three species (Fig. 3b).

Non-metric Multi-Dimensional Scaling (nMDS) analysis of normalized data for all pigments retrieved by spectrophotometry (Chl *a*, Chl *b*, Chl *c*, Chl *d*, Total Chlorophyll and Carotenoids) showed no grouping according to SSC treatment (Fig. 3c). The only exception was *Carteriospongia foliascens* exposed to 100 mg L^−1^, which grouped closer together, consistent with patterns observed for Chl *a*. PERMANOVA analysis confirmed significant differences between treatments in *Carteriospongia foliascens*, but also in *Cliona orientalis*, with subsequent pair-wise testing showing main differences between low and high SSCs (Table 2c).

### Microbial community analysis

**Figure 4:**
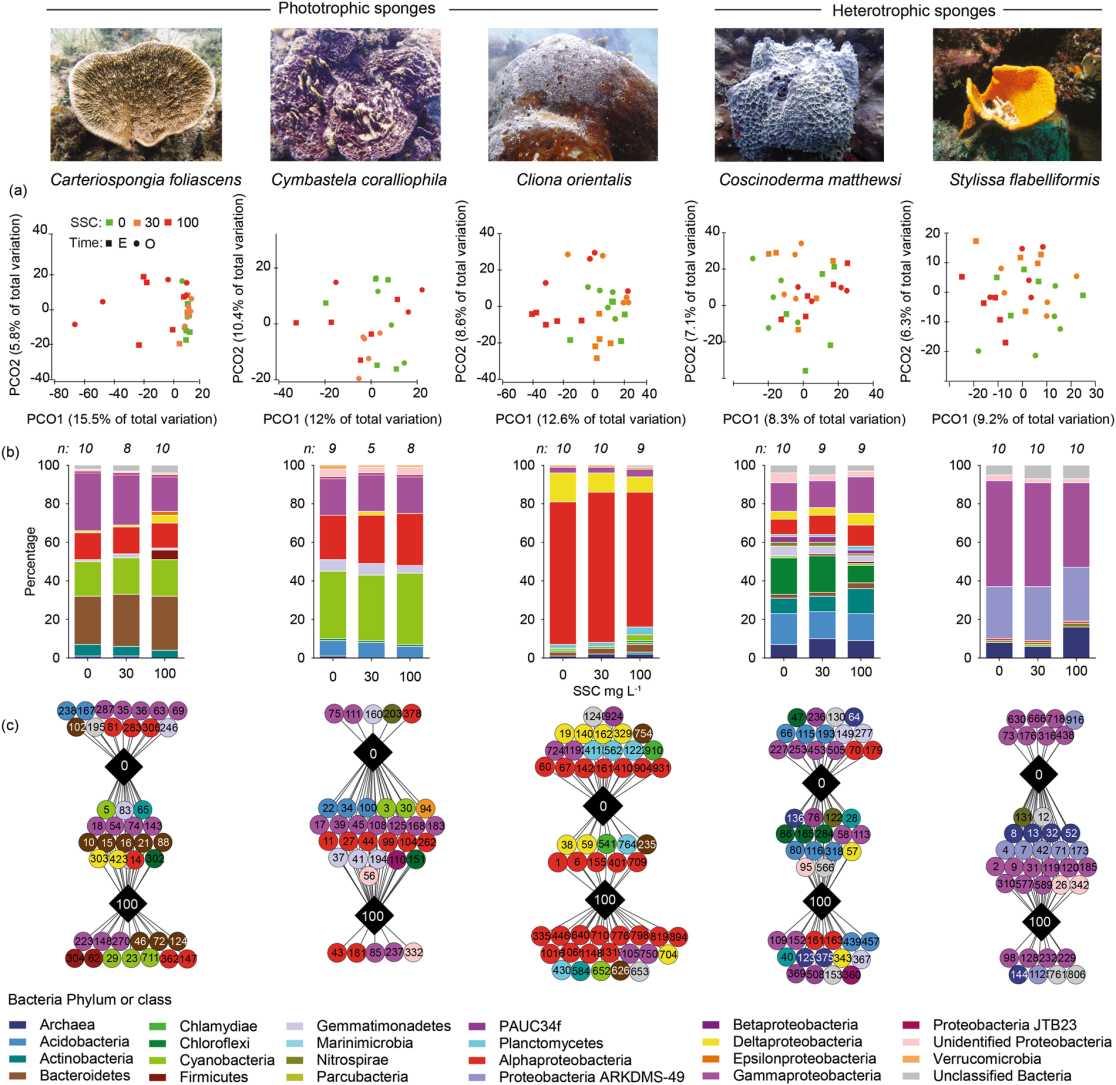
Microbial responses to SSCs. (**a**) Principal coordinate analysis plots for all species and treatments after the 28 d experimental period (E) and 14 d observational period (O), (**b**) Average relative abundance of each bacteria phylum (and class for *Proteobacteria*) using OTUs representing greater than 1% of the community for each treatment across both sampling times, (**c**) Cytoscape networks of the microbiome community in all species at 0 and 100 mg L^−1^ of SSC across both sampling times. Circles correspond to different OTUs (with OTU numbers) and colours relate to their phylum or class level in the case of Proteobacteria.

A total of 9,530,650 high quality 16 S rRNA gene amplicon sequences were recovered from the 5 sponge species (n = 200 individual samples) and 9 seawater samples. Each sponge species maintained a unique microbial community (Table 3a, Fig. 4, see also Supplementary Fig. S5 and Table S2) that was distinct from the seawater microbiome (Table 3b, Supplementary Figs S5 and S6). Aquarium acclimation (for 4 weeks) did not affect the sponge-associated microbial community of any species (Table 3a) as field controls were not significantly different from Time 0 controls (i.e. samples collected after the acclimation period, right before starting the exposure to sediments) (Table 3a). However, both field/time 0 controls showed significant differences with samples at the end of the experimental/observational period in all species (Table 3a). Elevated SSC did not have a major impact on the overall composition of the sponge microbiome at the phyla level (Fig. 4b) and no significant differences were detected at the OTU level (97% sequence similarity) for any species (Table 3c). In addition, no clear groupings were observed in the ordination (Fig. 4a). The only exception to this microbial stability was in *Carteriospongia foliascens*, where samples exposed to 100 mg L^−1^ exhibited greater dispersion across the ordination (Fig. 4a). *Cliona orientalis* also showed greater separation between samples at the end of the experimental and observational periods, particularly in the 30 and 100 mg L^−1^ treatments (Table 3c, Fig. 4a).

**Table 3:** PERMANOVA analyses of the sponge-associated microbiome with (A) species and time as factors, (B) source as factor (sponge host vs. seawater) and, (C) SSC and time (nested to SSC) as fixed factors for all five sponge species.

Source	*df*	MS	Pseudo-*F*	*P* (perm)
A)				
Species	4	73421	34.606	0.0001
Time	3	4680	2.2062	0.0001
Species × Time	1	3344	1.5765	0.0001
Residuals	1	2121		
Pair-wise Tests				
CAR:F ≠ E(*P* < 0.005); CYM: F ≠ E,O(*P* < 0.005); CLI:F,T0 ≠ E ≠ O(*P* < 0.005); COS: F,T0 ≠ E,O(*P* < 0.05); STY: F,T0 ≠ E,O(*P* < 0.05)				
B)				
Source	1	26923	6.3132	0.0001
Residuals	1	4269		
C)				
*Carteriospongia foliascens*				
SS	2	3447	1.54	0.1604
Time (SS)	3	2239	1.0156	0.3855
Residuals	2	2204		
*Cymbastela coralliophila*				
SS	2	1474	1.0237	0.4304
Time (SS)	2	1427	1.1049	0.199
Residuals	1	1291		
*Cliona orientalis*				
SS	2	4458	1.0368	0.3809
Time (SS)	3	4302	1.7738	0.0001
Residuals	2	2425		
*Coscinoderma matthewsi*				
SS	2	3362	1.2413	0.0682
Time (SS)	3	2709	1.0787	0.1323
Residuals	2	2511		
*Stylissa flabelliformis*				
SS	2	1984	1.1119	0.2005
Time (SS)	3	1784	1.0984	0.1205
Residuals	2	1624		

In pair-wise tests, F: field control, T0: time 0 control, E: sampling after the 28 d experimental period, O: sampling after the 14 d observational period; CAR for *Carteriospongia foliascens*, CYM for *Cymbastela coralliophila*, CLI for *Cliona orientalis*, COS for *Coscinoderma matthewsi* and STY for *Stylissa flabelliformis*; 0, 30 and 100 mg L^−1^ within SSC.

Network analyses of the 30 most discriminatory operational taxonomic units (OTUs) for the control (0 mg L^−1^) and highest SSC (100 mg L^−1^) in each species revealed phylogenetically diverse and stable core microbiomes (i.e. the component shared between all samples) as well as treatment specific OTUs (Fig. 4c, Supplementary Table S2). In each species, the number and taxonomy of OTUs observed exclusively in control samples was generally balanced by the number and taxonomy of new OTUs that were observed only in sediment treated samples. A notable exception however was the recruitment of 3 novel *Cyanobacteria* OTUs in *Carteriospongia foliascens* within samples from the high SSC. Also of interest was the finding that core *Cyanobacteria* OTUs in the two photosynthetic species *Carteriospongia foliascens* and *Cymbastela coralliophila* are not disrupted by sediments, even at the highest SSC.

## Discussion

Establishing dose-response relationships relies on identifying the significance and relative importance of different cause-effect pathways. As part of a sequence of experiments, and a wider investigation into the effects of sediment deposition, light attenuation and elevated SSC (alone and in combination)^38, 44^, we exposed five sponge species spanning a range of morphologies and nutritional modes to elevated SSCs for a chronic (28 d) period. Light levels were kept constant across the different treatments to isolate SSC as the primary variable. The SSCs used in this study (range 0–100 mg L^−1^) were designed to bracket SSCs measured during a recent large scale capital dredging project in NW Australia (the Barrow Island project, see ref. 4). During this project, at 4 shallow water sites (~5–11 m depth) located <~0.5 km from the dredging, the 95^th^ percentile (*P*_95_) of SSCs over a 30 d running mean period was 16.0 mg L^−1^ (range: 12.9–20.9) during the dredging phase as compared to 2.0 mg L^−1^ (range: 1.5–3.1) before dredging. Exposure of the sponges to SSCs ≥23 mg L^−1^ which corresponded to the 30 and 100 mg L^−1^ nominal treatments, for 28 d had an overall negative effect on sponge physiology, including decreases in lipid content, suggesting that sponge feeding behaviour may be compromised. High levels of SSC also resulted in negative growth, decreased respiration rates and caused significant necrosis and mortality within the cup sponge *Carteriospongia foliascens* and, to a lesser extent, the massive sponge *Coscinoderma matthewsi*. The high levels of *Carteriospongia foliascens* mortality in response to elevated SSCs supports previous research proposing this species as a sensitive bioindicator for assessing impacts from the dredging related pressure of light attenuation^44^. *Cliona orientalis* was also negatively affected by high SSC, although it demonstrated a capacity for rapid recovery. LC_50_ and LC_10_ levels could only be calculated for *Carteriospongia foliascens* and were 40.61 and 21.51 mg L^−1^, respectively. On the other hand, exposure to SSCs ≤ 10 mg L^−1^ was tolerated by most species over the experimental period. These water quality values cannot be used for impact prediction and management purposes in isolation, as the SSCs are likely to have profound effects on light attenuation (see refs 4, 40 and 41) which is also known to significantly affect some sponge species used in this study^44^.

In general, respiration rates decreased with elevated SSC, consistent with responses reported for the deep sea sponge *Geodia barretti* following sediment exposure^45, 46^. *Cliona orientalis*, *Cymbastela coralliophila* and *Stylissa flabelliformis* were able to metabolically recover within two weeks when exposed to the lowest sediment treatment (3 mg L^−1^); however, a greater recovery time is likely required at higher SSC. In contrast to the other species, respiration rates in the massive species *Coscinoderma matthewsi* increased significantly at 100 mg L^−1^, a trend that has also been reported in other heterotrophic massive species following short term exposure to elevated SSC^47^. However, the higher respiration rates in *Coscinoderma matthewsi* may also be a result of stress associated with oxygen depletion within the experimental respiration chambers^48^. Hence, due to the wide range of species-specific responses, it is difficult to generalise about how sponge respiration rates will respond to elevated SSCs.

Sponges are highly efficient filter-feeders, processing large amounts of water from which they efficiently remove bacteria and nutrients^13, 14^. Elevated SSCs from natural events or anthropogenic activities such as dredging are a natural hazard to the filter feeding mode of nutrition^49, 50^. Different sponge species have evolved different strategies to avoid excessive sediment intake and prevent clogging of their aquiferous systems. Sponges can slow their pumping activity^33, 34, 46^ and accumulate sediments in pockets that can be subsequently expelled with mucus secretions (reviewed in refs 32 and 39). Some sponges such as *Cliona orientalis*, are also capable of entirely closing their oscula (excurrent opening) in response to sediment deposition^36^. Oscular closure can decrease and entirely arrest sponge pumping within hours^36^ which would result in decreased respiration rates over very short time scales^46, 51^.

Different sediment removal mechanisms are likely to have very different energetic demands. However, all strategies are thought to contribute to a reduction in the food-retention efficiency of the sponges’ aquiferous system^47, 52^ which may compromise energy stores in the long term. Lipids are essential to an organism’s physiological processes as they are a major source of metabolic energy and are often considered the most effective energy store in marine ecosystems^53, 54^. Lipids can be broadly grouped into storage and structural functions, with storage lipids predominantly being used as energy reserves and both categories playing a significant role in stress tolerance^55–57^. A decrease in lipids has previously been associated with lower levels of energy storage and lower survival rates in corals^58, 59^ and sponges^16^. However, in this study, no significant decreases in total lipid reserves were observed in sponges exposed to high SSC. Phototrophic sponges may have the potential to compensate their energy intake through photosymbiosis. For instance, the variable lipid content in the phototrophic *Cliona orientalis* across treatments likely reflects the flexible feeding strategy of this species including an ability to switch to heterotrophic feeding, as evidenced by its survival during extended periods of darkness^44^ and consistent with results for other species of the same genera^60^. The increase in total lipids in *Cliona orientalis* in the 100 mg L^−1^ treatment may result from an increase of the photosynthetic dinoflagellates *Symbiodinium* sp. in an attempt to increase phototrophic feeding, as corroborated by histological results. In a similar way, *Symbiodinium* sp. living in shallow water corals have been reported to supply host polyps with an excess of energy-rich lipids^61, 62^. In *Cliona orientalis* these energy stores were likely used during the observational period to aid recovery from bleaching and tissue regression and to increase choanocyte chamber density; although a longer recovery time may be necessary to return total lipids to control levels.

As light was compensated across treatments to maintain equivalent daily light integrals and intensities irrespective of SSC, no direct effects were detected of SSC on maximum quantum yields or Chl a concentrations, until disruption of the holobiont due to temporary (e.g. *Cliona orientalis*) or permanent bleaching (e.g. *Carteriospongia foliascens*). Hence, our results suggest that the health and photosynthetic efficiency of the photosymbionts is unaffected by high SSC in the absence of light attenuation. Interestingly though, increased chlorophyll d in samples of *Carteriospongia foliascens* and *Cymbastela coralliophila* exposed to 100 mg L^−1^ suggested an increase in some Chl d-containing Cyanobacteria^63^, providing further support for increased reliance on phototrophic feeding under high SSC. Previous research has also shown that the presence of photosymbionts often influences the composition of the host associated microbiome^64^ hence any SSC induced change in photosymbiosis may have flow-on effects for the entire sponge microbiome.

Overall, sponge associated microbiomes were not significantly affected by SSC in any species, although minor differences were observed in *Carteriospongia foliascens* and *Cymbastela coralliophila*. In particular, under high SSC (100 mg L^−1^) an increase in *Firmicutes*, *Deltaproteobacteria* and *Epsilonproteobacteria* was evident which is consistent with numerous reports of microbial shifts associated with stressed and diseased sponges^65–67^. Interestingly, novel *Cyanobacteria* OTUs inhabited *Carteriospongia foliascens* and *Cliona orientalis* exposed to high SSC, indicating a potential mechanism for acclimation to a high SSC environment. However, while some species may have the ability to successfully alter the composition of their microbial community under different environmental conditions^44, 68, 69^, species with intimate and/or potentially obligate symbioses can be adversely impacted by disruption of their microbiome^66, 67^ or loss of symbiotic function^70^. The rapid deterioration in health of *Carteriospongia foliascens* under conditions of high SSC following the microbiome shift is consistent with its documented intimate reliance on a highly specialised microbial community^44^.

Our results and previous studies suggest that most sponges have well developed mechanisms to survive periods of elevated SSC, however extended exposure to high SSC has significant adverse effects on sponge metabolism^34, 45, 47^. Some species appear to have plasticity in modifying their aquiferous system^71, 72^, including the density of their choanocyte chambers^73^ depending on environmental conditions. Shifts from phototrophy to heterotrophy depending on irradiance have been also described in numerous sponge species^16, 27, 74^. Alternatively, mixotrophic sponges may increase their reliance on phototrophic nutrition as observed in the current study. Our results also suggest that discolouration (i.e. bleaching) and necrosis of sponge tissue are effective bioindicators for dredging related stress.

In this study, the isolation of SSC from other dredging-related factors (i.e. sedimentation and light attenuation) allowed us to identify a specific cause:effect pathway and determine lethal and sub-lethal SSC for sponges^4^, including LC_50_ and LC_10_ levels for *Carteriospongia foliascens* (i.e. 40.61 and 21.51 mg L^−1^, respectively). In conjunction with previous research on the impacts of light attenuation^44^, these thresholds can now be used in water quality monitoring programs to alert dredging proponents to levels of light reduction and SSC that, if continued, could detrimentally impact sponge populations. In combination with sediment plume and light attenuation models, these results can be used to predict the likely effects of dredging (i.e. at the EIA stage) and to establish impact zones. By modelling different dredging scenarios (i.e. volume of material dredged, tidal phase, over flow options, etc.) the information could also be used to identify optimal dredging scenarios that minimise the likelihood and extent of impact.

Here we show that exposure to high SSC (≥23 mg L^−1^) for extended periods (28 d) has a negative effect on sponge feeding behaviour with associated depletion of energy reserves. However, while ≤10 mg L^−1^ for <28 d seems tolerable by most species and could be established as a prudent sub-lethal threshold in adult sponges, it is expected that the combined effect of SSC with low light availability and sediment smothering will increase the negative effects on sponges under realistic field conditions. These experimental results will assist regulators and environmental managers in reducing risks from dredging development although experimental research combining sedimentation, SSC and light attenuation is required before final thresholds can be derived for dredging impacts on sponges.

## Methods

### Sample collection

To assess impacts across nutritional modes, this study used the phototrophic sponges *Carteriospongia foliascens* (Pallas, 1766), *Cymbastela coralliophila* (Hooper & Berquist, 1992) and *Cliona orientalis* (Thiele, 1900) and the heterotrophic sponges *Coscinoderma matthewsi* (Lendenfeld, 1886) and *Stylissa flabelliformis* (Hentschel, 1912)^18, 23, 25, 38, 75^. All species are common throughout the Indo-Pacific, including the east and west coasts of tropical Australia. Sponges were collected from 3–15 m depth from the Palm Islands, Great Barrier Reef (GBR) (Supplementary Table S3). *Cliona orientalis* is an encrusting sponge that bioerodes coral, so cores of *Cliona orientalis* were air-drilled from dead colonies of *Porites* sp. ensuring >2 cm of coral substrate below the sponge. For all species, sponges were cut into similar sized explants (~5 × 5 cm), and acclimated under natural light in flow-through seawater for 4 weeks until fully healed.

### Experimental set up

The SSC exposure was performed in the National Sea Simulator (SeaSim) at the Australian Institute of Marine Science (AIMS, Townsville) using square 115L clear PVC acrylic tanks with an inverted pyramid at the base. Water within the tanks was circulated by a magnetic drive centrifugal pump that collected water from the top of the tank and forced flow up from the centre point of the inverted pyramid at the base. Sponges were placed on a gridded, false bottom floor 20 cm from the top of the tank and a second pump (VorTech™ MP10, EcoTech Marine, PA, US) was placed in the tank at the same height as the sponges to aid in water circulation. The seawater in experimental tanks was set to 27 °C, representing the temperature at the time of sponge collection.

Sponge explants were exposed to 5 nominal SSCs of 0 (i.e. experimental control), 3, 10, 30 and 100 mg L^−1^ and the tanks were supplied with a continuous inflow of 5 µm filtered seawater at a rate of 400 mL min^−1^, providing ~6 complete turnovers of seawater in each tank per day. Each treatment level had 3 replicate tanks containing 3–4 sponge replicates per species. To replace sediment lost from the tanks because of the continuous flow through of filtered seawater, new sediment was periodically introduced by small volumes of sediment slurry (~8 g L^−1^) from an adjacent 500 L stock suspension. SSCs within each tank were monitored and controlled from turbidity readings (as nephelometric turbidity units, NTUs) using Turbimax CUS31 (Endress and Hauser, Germany) nephelometers connected to a programmable logic controller (PLC) system (see below). NTUs were converted to SSCs (as mg L^−1^) by applying sediment specific algorithms determined gravimetrically (filtration of SSCs through 0.4 µm (nominal pore size) polycarbonate filters and calculating dry weight of the filters). The PLC system monitored NTU in each tank and controlled the SSCs by episodically opening and closing solenoid valves connected to the stock tank. Triplicate 250 mL water samples were collected from each tank weekly and the SSCs determined gravimetrically. Differences in SSC between treatments throughout the experiment were studied at each sampling day separately with a one-way analysis of variance (ANOVA) using treatment as the fixed factor. Data was log-transformed to achieve homogeneity of variances and normality when required.

The sediment used in this experiment was selected after an initial comparison between inshore sediments (siliclastic sediment collected subtidally from Onslow, Western Australia)^38^ and offshore sediments (calcareous sediment collected from the lagoon of Davies Reef, a mid-shelf reef centrally located in the GBR, Queensland; S 18° 49.354′ E 147°38.253′). Similar responses were observed in sponges from comparative dosing for both sediments. For comparative purposes with other experiments, the calcareous sediment from Davies Reef was selected. Most importantly, all sediments were ground to ~30 µm (with 80% of the sediment 3–65 µm) with a rod mill grinder and measured using laser diffraction techniques (Mastersizer 2000, Malvern instruments Ltd, UK). Hence, the sediments used were predominantly silt-sized, typical for dredge plumes, to ensure environmental relevance. Tanks were illuminated by AI Hydra FiftyTwo™ HD LED lights (Aquaria Illumination, IA, US) on a 13:11 h L:D cycle. The light regime was designed to simulate daily conditions on the reef ^41, 76^, and made up of a 6 h period of gradually increasing light in the morning (06:00–12:00 h), an hour of constant illumination at 200 µmol photons m^−2^ s^−1^, and a 6 h period of gradually decreasing light in the afternoon (13:00–19:00 h). Over the course of the day the sponges experienced a daily light integral (DLI) of 5 mol photons m^−2^.

In order to isolate the effect of SSC from the associated light attenuation that would occur under different SSCs, light intensities were adjusted between the tanks using a Li-250A light meter (LI-COR Biosciences, NE, US) at the grid level and adjusting the light intensity provided by the LED lights accordingly to ensure that all sponges received the same DLI regardless of SSC treatment. The surface of all sponges was also gently brushed each day to remove any deposited sediment that could negatively affect the sponges. The SSC experiment was conducted for a 28 d ‘experimental’ period, followed by a 14 d ‘observational’ period in clear water (0 mg L^−1^). The length of the experiment was selected to simulate chronic conditions and to facilitate comparison with the 30-days running means for dredging-related turbidity events from 3 major capital dredging programs in North Western Australia^41^.

### Studied parameters and statistical analyses

The effect of SSC on the sponge holobiont was determined using a suite of response variables, with a particular focus on changes in sponge feeding strategies, including changes in sponge photosymbionts, composition of the sponge microbial community and growth. To obtain baseline data on sponge health, 6 extra individuals were processed for each species immediately after collection (field controls) and after aquarium acclimation (t = 0 controls). From the initial 10 individuals stocked per species and treatment, 5 individuals were sampled (and removed from the experiment) for ‘destructive’ response variables (i.e. pigments, lipids and microbial community analyses) at the end of the experimental period and the remaining 5 individuals were sampled at the end of the observational period, in order to avoid negative effects on sponge health due to sub-sampling of the same individuals. Unless otherwise stated, statistical analyses were performed and graphs prepared using the software R v. 3.1.0 and SigmaPlot v.11.0 (Systat Software Inc.).

Linear mixed models using tank as a random factor and treatment as a fixed factor were fitted by residual maximum likelihood (REML) for data pertaining to relative growth rates, necrosis, lipid content, respiration, histology, chlorophyll fluorescence and total pigments at the end of the exposure and observational periods for each species, separately. An analysis of variance (ANOVA) table was generated for each model and Tukey’s post–hoc multiple comparisons were performed to compare treatment levels for each model^77^.

### Growth and necrosis

Initial and final thickness (in *Carteriospongia foliascens*, *Cymbastela coralliophila* and *Stylissa flabelliformis*), height (in *Coscinoderma matthewsi*), or sponge tissue depth inside coral cores (in *Cliona orientalis*), of each sponge (±0.1 mm) was measured with callipers as a 2-dimensional proxy for growth. Three measurements per sponge were averaged to calculate percentage change throughout the experimental and subsequent observational period. Changes in size, partial mortality (i.e. necrosis, assessed through species-specific changes in tissue characteristics, such as changes towards brown/black colour, absence of a healthy pinacoderm and exposure of fibres and skeleton) and loss of photosynthetic symbionts (bleaching) was recorded weekly using a digital camera (Canon S120) with underwater housing and analysed using image analysis software (ImageJ^78^). Relative growth rates were calculated as the logarithm of the final measure divided by the initial measure, based on both thickness of sponges and their surface area. Percent mortality per tank (n = 3 per treatment) was fitted to nonlinear regression curves using the program Prism v7.01 (GraphPad Software Inc, CA, US). Regression curves were used to calculate the lethal concentrations (LC) of SSC at which 50% (LC_50_) and 10% (LC_10_) of the population died. The models were constrained between 0 and 100 with F values set at 50 and 10, for LC_50_ and LC_10_, respectively. The curve was tested for normality of the residuals and a replicate test was applied to assess goodness of fit. Symmetric, asymptotic confidence intervals were calculated for the LC values.

### Lipid analysis

The concentration of total lipids in sponge tissue was measured over time to assess whether SSC interferes with sponge feeding capability. Samples were analysed from the field controls and at the start of the experiment and at the end of the experimental and observational periods for samples exposed to the 0, 30 and 100 mg L^−1^ SSCs. Approximately 3 cm^3^ of sponge tissue was excised, wrapped in aluminium foil to prevent plasticizer contamination and immediately frozen in liquid nitrogen. Lipids were extracted from approximately 100 mg of freeze-dried ground sample as described in ref. 79 and following modifications in ref. 80, with total lipid content reported as percentage biomass based on a dry weight conversion factor.

### Respiration rates

Changes in sponge respiration rates were measured throughout the experimental and observational period. Samples were dark adapted for a minimum of 30 min before being transferred to 600 mL respiration chambers where they were incubated for 30 min at 27 °C. Constant mixing within the chamber was achieved using a magnetic stir bar and a submergible battery-operated platform. An HDQ30D flexi meter (HACH LDO™, CA, US) was used to take three initial readings (i.e. initial O_2_) and to measure the mg L^−1^ of O_2_ and the % O_2_ in each chamber at the end of the incubation. The final oxygen concentration inside the chamber did not drop below 85% saturation in most cases. To control for microbial community respiration, a chamber containing only environmental water (blank) was incubated under identical conditions.

### Histology

Changes in the structure of the aquiferous system, in particular the number of choanocyte chambers, was examined from histological sections of samples collected at the end of the 28 d experimental period for the 0 and 100 mg L^−1^ treatments. Samples were fixed for 6 weeks in FAACC fixative (10 mL 40% formaldehyde, 5 mL glacial acetic acid, 1.3 g calcium chloride dehydrate, 85 mL water), and then transferred to 70% ethanol and stored at room temperature. Samples were dehydrated through a graded series of ethanol, embedded in paraffin, sectioned to 5 µm and stained using Mayer’s Haematoxylin, and Young’s Eosin Erythrosine. Samples were visualized and photographed using an Axioskop 2 plus Microscope and AxioCam MRC5 Digital Camera (Carl Zeiss Microscopy, LLC, US). Choanocyte chambers were only quantified for *Cliona orientalis* as the presence of spicules in all other species resulted in poor quality sections that precluded accurate choanocyte chamber quantification. In *Cliona orientalis*, five fields of view (FOV) per section were examined to quantify the total number of choanocyte chambers at 200×. Choanocyte chambers were identified as circular rings of cells^73^ and chamber density was averaged across the 5 FOVs to provide a mean choanocyte chamber density per sponge.

### Chlorophyll fluorescence

Changes in photosynthetic capacity (maximum quantum yield) of the sponge’s phototrophic symbionts were measured with a Diving-PAM (pulse amplitude modulation) chlorophyll fluorometer (Heinz Walz GmbH, Effeltrich, Germany) for *Carteriospongia foliascens*, *Cymbastela coralliophila* and *Cliona orientalis* as described in ref. 44. Briefly, maximum quantum yield (*F*_v_/*F*_m_)^81^ measurements were obtained from dark-adapted sponges at weekly intervals throughout the experimental and observational periods.

### Pigment analysis

Pigment analyses were performed on ~0.2 g of tissue from all phototrophic sponges (*Carteriospongia foliascens*, *Cymbastela coralliophila* and *Cliona orientalis*) at the end of the experimental and recovery periods. Pigments from samples incorporating pinacoderm and mesohyl regions were extracted and analysed as described in ref. 44 and standardized to sponge wet weight. The concentration of Chlorophyll *a* (hereafter Chl *a*) was used as a proxy for changes in photosymbiont health/activity (i.e. bleaching)^18^. Differences between Chl *a* at the end of the 28 d experiment and after the 14 d recovery phase were assessed with a t-test for every treatment and species, separately. Pearson correlations were performed between all the studied pigments. All pigments retrieved by spectrophotometry (i.e. chlorophylls *a*, *b*, *c*, *d*, Total chlorophylls and carotenoids) were used to build resemblance matrices based on normalized data for each species, separately. Non-metric Multi-Dimensional Scaling (nMDS) plots were created using Euclidean distances. Two factors were determined (i.e. SSC and sampling time, nested to SSC) and examined by PERMANOVA (Permutational multivariate ANOVA based on distances). All multivariate analyses were performed using Primer 6 (Primer-E Ltd, UK).

### Microbial community analysis

Microbial community composition was assessed using Illumina amplicon sequencing of the 16 S rRNA taxonomic marker gene, for field controls, t = 0 controls and at the end of the experimental and observational periods for sponges exposed to 0, 30 and 100 mg L^−1^ SSC. All samples were immediately frozen in liquid nitrogen and subsequently stored at −80 °C. Water was also collected from each tank at the time of sampling to facilitate a direct comparison with microbes present in the surrounding environment. DNA was extracted from ~0.2 g of sponge tissue using the PowerPlant® Pro DNA Isolation Kit (MoBio Laboratories, CA, US) according to the Manufacturer’s protocol. Microbial communities in seawater were filtered and DNA was extracted as previously described^82^. Sequencing of the 16 S rRNA gene was performed at the Australian Centre for Ecogenomics using primers 515f and 806r and the Illumina HiSeq2500 platform. Sequence data was deposited at the NCBI under the accession number SRP080228.

Amplicon sequence data was processed in Mothur v.1.35.1^83^ according to the MiSeq standard operating procedure^84^. Briefly, demultiplexed fastq paired-end reads were first quality-filtered and assembled into contigs (make.contigs and screen.seqs: maxambig = 0, maxhomop = 8, minlength = 100, maxlenght = 292). Aligned reads were reduced to non-redundant sequences and chimeric sequences were detected using Uchime^85^. Aligned sequences were phylogenetically classified based on the Silva reference file v.123, and all unassigned sequences removed (taxon = Chloroplast-Mitochondria-unkown-Eukaryota). Samples with low read numbers were eliminated from the dataset and remaining samples were sub-sampled to 10385 sequences. Pairwise distances were calculated and used for clustering and OTU assignment and OTUs were further classified based on the SILVA v.123 taxonomy.

OTU data was normalised to account for sampling depth and then square-root transformed to reduce the effect of abundant OTUs. Bray-Curtis distance matrices were constructed and visualised using non-metric multidimensional ordinations (nMDS) and principal coordinates analyses (PCO). Permutational analysis of variance (PERMANOVA, using 9999 permutations) was used to determine significant differences in microbial communities. All multidimensional statistical analyses were performed in Primer 6/PERMANOVA. Similarity Percentage (SIMPER) analysis was used to determine the OTUs that contribute to the differences between 2 SSC (0 and 100 mg L^−1^) for each phototrophic species, separately. The 30 OTUs with the most discriminating power from the SIMPER analysis were used to create networks on Cytoscape 3.2.0^86^.

## Electronic supplementary material

Supplementary Information

Supplementary Dataset 1
