# Supplementary material for: Effects of suspended sediments on the sponge holobiont with implications for dredging management
Source: Sci Rep. 2017 Jul 10;7:4925. doi: 10.1038/s41598-017-05241-z (PMC5504051; doi:10.1038/s41598-017-05241-z)

Supporting Online Material

Effect of suspended sediments on the sponge holobiont with implications for dredging management

Mari-Carmen Pineda[1,2*], Brian Strehlow[1,2,3], Miriam Sternel[4], Alan Duckworth[1,2], Ross Jones[1,2] and Nicole S. Webster[1,2]

[1] Australian Institute of Marine Science (AIMS), Townsville, QLD and Perth, WA, Australia
[2]Western Australian Marine Science Institution, Perth, WA, Australia
[3]School of Plant Biology and Centre for Microscopy Characterisation and Analysis: University of Western Australia, Perth, WA, Australia
[4]University of Bremen, Bremen, Germany

*Corresponding author:

Mari-Carmen Pineda

Australian Institute of Marine Science, PMB3, Townsville, QLD, 4810, Australia

E-mail: mcarmen.pineda@gmail.com.

Tel.: +61 7 4753 4522, fax: +61 7 4772 5852

Table S1. ANOVA tables and summaries of linear mixed models examining the effects of treatment on relative growth rate based on surface area (not relevant for *Cliona orientalis*), for each species separately, at the end of the experimental and observational periods. Tukey tests have been performed for significant pairwise multiple comparisons.

		Carteriospongia foliascens		*Cymbastela coralliophila*		*Coscinoderma matthewsi*		*Stylissa flabelliformis*	
Source	*df*	*F*	*P*	*F*	*P*	*F*	*P*	*F*	*P*
Experimental phase									
Treatment	4	14.315	0.060	5.795	0.04	6.741	0.03	2.865	0.1394
Error	20								
Tukey		0>3,10,30>100		0,3,>30,100		0>3,10,100			
Observational phase									
Treatment	4	11.646	0.01	8.20	0.020	4.65	0.061	3.704	0.092
Error	20								
Tukey		0,3>10,30,100		0,3>30,100					

Table S2. Similarity Percentage Analysis (SIMPER) for 30 most significant OTUs driving differences between 0 and 100 mg L^{-1} SSC.

**OTU**	**Average relative abundance (%)**		**Contribution (%)**	**Taxonomic ID**
	**0 mg L^{-1}**	**100 mg L^{-1}**		
Carteriospongia foliascens				
Otu000005	2.53	2.82	1.04	Cyanobacteria
Otu000010	1.76	1.4	0.82	Bacteroidetes
Otu000015	1.35	0.91	0.68	Bacteroidetes
Otu000062	0	1.16	0.64	Firmicutes
Otu000035	1.3	0.52	0.62	Gammaproteobacteria
Otu000023	0.8	0.9	0.6	Cyanobacteria
Otu000029	0.68	0.95	0.59	Cyanobacteria
Otu000025	0.97	0.5	0.56	Alphaproteobacteria
Otu000036	1.11	0.44	0.54	Gammaproteobacteria
Otu000018	1.44	0.74	0.53	Gammaproteobacteria
Otu000014	1.69	1.17	0.53	Alphaproteobacteria
Otu000016	1.63	1.45	0.5	Bacteroidetes
Otu000021	1.26	1.42	0.46	Bacteroidetes
Otu000063	0.88	0.6	0.42	Gammaproteobacteria
Otu000065	1.05	0.71	0.41	Actinobacteria
Otu000046	0.32	0.88	0.41	Bacteroidetes
Otu000069	0.82	0.25	0.41	Gammaproteobacteria
Otu000050	0.75	0.37	0.4	Cyanobacteria
Otu000103	0	0.68	0.39	Bacteroidetes
Otu000081	0.85	0.59	0.39	Alphaproteobacteria
Otu000102	0.79	0.27	0.37	Bacteroidetes
Otu000304	0	0.66	0.37	Firmicutes
Otu000096	0.43	0.46	0.36	Gammaproteobacteria
Otu000072	0.79	0.74	0.35	Bacteroidetes
Otu000084	0.61	0.41	0.35	Alphaproteobacteria
Otu000082	0.48	0.39	0.35	Bacteroidetes
Otu000148	0.35	0.78	0.34	Gammaproteobacteria
Otu000054	0.87	0.73	0.33	Gammaproteobacteria
Otu000074	1.02	0.56	0.32	Gammaproteobacteria
Otu000362	0	0.62	0.32	Alphaproteobacteria
Cymbastela coralliophila				
Otu000022	1.97	0.8	1.48	Acidobacteria
Otu000030	1.1	1.26	1.47	Cyanobacteria

Otu000011	2.33	2.93	1.42	Alphaproteobacteria
Otu000044	1.49	0.62	1.2	Alphaproteobacteria
Otu000043	0.6	0.97	1.16	Alphaproteobacteria
Otu000041	1.1	0.63	1.11	Gemmatimonadetes
Otu000033	0.92	0.63	1.08	Cyanobacteria
Otu000017	1.89	1.6	1.08	Gammaproteobacteria
Otu000092	0.82	0.84	1	Cyanobacteria
Otu000068	0.91	0.52	0.98	Alphaproteobacteria
Otu000003	5.1	5.31	0.97	Cyanobacteria
Otu000056	0.93	1.22	0.93	Unidentified Proteobacteria
Otu000037	1.11	1.24	0.93	Gemmatimonadetes
Otu000202	0.24	0.66	0.83	Alphaproteobacteria
Otu000075	1.07	0.68	0.8	Gammaproteobacteria
Otu000027	1.63	1.6	0.8	Alphaproteobacteria
Otu000184	0.3	0.57	0.78	Alphaproteobacteria
Otu000034	0.94	1.38	0.76	Acidobacteria
Otu000207	0.68	0.48	0.74	Alphaproteobacteria
Otu000160	0.77	0.41	0.72	Gemmatimonadetes
Otu000111	0.93	0.64	0.72	Gammaproteobacteria
Otu000045	0.96	1.17	0.72	Alphaproteobacteria
Otu000097	0.47	0.61	0.71	Alphaproteobacteria
Otu000094	0.93	0.78	0.69	Verrucomicrobia
Otu000087	0.4	0.49	0.68	Gammaproteobacteria
Otu000104	0.81	0.93	0.67	Alphaproteobacteria
Otu000125	0.73	0.83	0.66	Gammaproteobacteria
Otu000039	1.28	1.17	0.65	Gammaproteobacteria
Otu000181	0.38	0.7	0.64	Alphaproteobacteria
Otu000201	0.57	0.34	0.63	Unidentified Proteobacteria
Cliona orientalis				
Otu000019	1.62	0.41	0.9	Deltaproteobacteria
Otu000060	1.22	0.34	0.59	Alphaproteobacteria
Otu000001	6.67	6.47	0.51	Alphaproteobacteria
Otu000038	1.09	0.67	0.48	Deltaproteobacteria
Otu000006	3.1	2.39	0.48	Alphaproteobacteria
Otu000059	0.68	0.87	0.39	Deltaproteobacteria
Otu000091	0.35	0.52	0.39	Alphaproteobacteria
Otu000067	0.77	0.18	0.36	Alphaproteobacteria
Otu000162	0.58	0.23	0.35	Deltaproteobacteria
Otu000248	0.09	0.6	0.31	Deltaproteobacteria
Otu000140	0.63	0.11	0.28	Deltaproteobacteria
Otu000142	0.6	0.12	0.28	Alphaproteobacteria
Otu000118	0.36	0.28	0.26	Alphaproteobacteria
Otu000329	0.47	0.08	0.22	Deltaproteobacteria
Otu000394	0.28	0.33	0.22	Gammaproteobacteria
Otu000255	0.02	0.43	0.21	Alphaproteobacteria
Otu000221	0.44	0	0.21	Alphaproteobacteria
Otu000410	0.43	0.09	0.21	Alphaproteobacteria
Otu000381	0.12	0.45	0.21	Cyanobacteria
Otu000254	0.38	0.06	0.2	Alphaproteobacteria
Otu000186	0.26	0.38	0.19	Plantomycetes
Otu000652	0.08	0.42	0.19	Cyanobacteria
Otu000077	0.3	0.25	0.19	Archaea
Otu000622	0.04	0.28	0.18	Alphaproteobacteria
Otu000182	0.29	0.15	0.18	Alphaproteobacteria
Otu000419	0.23	0.27	0.17	Verrucomicrobia
Otu000256	0.2	0.26	0.17	Archaea
Otu000475	0.17	0.32	0.17	Alphaproteobacteria
Otu000335	0.17	0.43	0.17	Alphaproteobacteria
Otu000420	0.23	0.21	0.16	Alphaproteobacteria
Coscinoderma matthewsi				
Otu000040	0.5	1.48	0.45	Actinobacteria

Otu000028	0.95	1.42	0.34	Actinobacteria
Otu000047	1.01	0.42	0.31	Chloroflexi
Otu000078	0.92	0.18	0.3	Chloroflexi
Otu000061	0.74	0.72	0.3	Gammaproteobacteria
Otu000106	0.78	0.47	0.27	Acidobacteria
Otu000066	0.93	0.45	0.26	Acidobacteria
Otu000057	0.84	1.2	0.23	Deltaproteobacteria
Otu000079	0.51	0.41	0.23	Gammaproteobacteria
Otu000132	0.65	0.49	0.22	Unidentified Proteobacteria
Otu000064	0.56	0.51	0.22	Archaea
Otu000053	0.43	0.46	0.21	Chloroflexi
Otu000130	0.75	0.35	0.2	Unclassified Bacteria
Otu000127	0.62	0.07	0.2	Chloroflexi
Otu000150	0.65	0.41	0.2	Actinobacteria
Otu000117	0.49	0.46	0.2	Acidobacteria
Otu000152	0.46	0.7	0.2	Gammaproteobacteria
Otu000070	0.67	0.4	0.2	Alphaproteobacteria
Otu000224	0.57	0.24	0.2	Chloroflexi
Otu000159	0.37	0.63	0.19	Alphaproteobacteria
Otu000049	0.2	0.65	0.19	Archaea
Otu000193	0.68	0.4	0.19	Acidobacteria
Otu000109	0.48	0.64	0.19	Gammaproteobacteria
Otu000174	0.39	0.42	0.18	Chloroflexi
Otu000218	0.42	0.57	0.18	Gammaproteobacteria
Otu000154	0.5	0.53	0.18	Acidobacteria
Otu000245	0.54	0.31	0.17	Chloroflexi
Otu000051	0.35	0.54	0.17	Chloroflexi
Otu000153	0.37	0.77	0.17	Unclassified Bacteria
Otu000086	0.89	0.57	0.17	Chloroflexi
Stylissa flabelliformis				
Otu000008	1.43	1.74	2.06	Archaea
Otu000013	0.65	1.68	1.63	Archaea
Otu000012	1.85	1.89	0.99	Unclassified Bacteria
Otu000032	0.67	1.03	0.88	Archaea
Otu000002	5.77	5.19	0.86	Gammaproteobacteria
Otu000071	0.44	0.75	0.84	Proteobacteria ARKDMS-49
Otu000052	0.55	0.99	0.8	Archaea
Otu000073	0.82	0.47	0.8	Gammaproteobacteria
Otu000004	3.65	3.54	0.77	Proteobacteria ARKDMS-49
Otu000031	0.83	0.94	0.69	Gammaproteobacteria
Otu000009	2.44	2.23	0.69	Gammaproteobacteria
Otu000120	0.63	0.54	0.67	Gammaproteobacteria
Otu000176	0.62	0.35	0.67	Gammaproteobacteria
Otu000026	1.39	1.21	0.66	Unidentified Proteobacteria
Otu000119	0.66	0.45	0.65	Gammaproteobacteria
Otu000098	0.52	0.54	0.65	Gammaproteobacteria
Otu000007	2.81	2.86	0.56	Proteobacteria ARKDMS-49
Otu000131	0.51	0.75	0.56	Nitrospirae
Otu000093	0.26	0.41	0.56	Nitrospirae
Otu000232	0.12	0.52	0.53	Gammaproteobacteria
Otu000128	0.37	0.41	0.5	Gammaproteobacteria
Otu000121	0.23	0.32	0.5	Gammaproteobacteria
Otu000144	0.32	0.58	0.49	Archaea
Otu000260	0.22	0.43	0.47	Gammaproteobacteria
Otu000315	0.05	0.44	0.46	Archaea
Otu000264	0.27	0.43	0.45	Unclassified Bacteria
Otu000175	0.14	0.34	0.45	Unclassified Bacteria
Otu000145	0.06	0.39	0.42	Proteobacteria ARKDMS-49
Otu000135	0.26	0.21	0.41	Archaea
Otu000173	0.54	0.41	0.41	Proteobacteria ARKDMS-49

Table S3. Sponge sampling details. List of species, morphologies, nutritional mode and sampling location.

Species Name (Author)	Functional Morphology	Primary Nutritional Mode	Sampling Location
Carteriospongia foliascens (Pallas, 1766)	Cup (wide cup)	Phototrophic [1]	Fantome Is. (Palm Is.) S 18°41.028' E 146° 30.706'
Cymbastela coralliophila Hooper & Bergquist, 1992	Encrusting (thick) Cup (table)	Phototrophic [2]	Pelorus Is. (Palm Is.) S 18°32.903' E 146° 29.172'
Cliona orientalis Thiele, 1900	Encrusting (bioeroding)	Phototrophic [3]	Pelorus Is. (Palm Is.) S 18°32.903' E 146° 29.172'
Coscinoderma matthewsi (Lendenfeld, 1886)	Massive	Heterotrophic [4]	Pelorus Is. (Palm Is.) S 18°32.903', E 146° 29.172'
Stylissa flabelliformis (Hentschel, 1912)	Erect (laminar)	Heterotrophic [5]	Pelorus Is. (Palm Is.) S 18°32.903' E 146° 29.172'

1. Ridley, C. P., Faulkner, D. & Haygood, M. G. Investigation of Oscillatoria spongeliae-dominated bacterial communities in four dictyoceratid sponges. *Appl. Environ. Microbiol.* **71,** 7366–75 (2005).
2. Cheshire, A. *et al.* Preliminary study of the distribution and photophysiology of the temperate phototrophic sponge Cymbastela sp. from South Australia. *Mar. Freshw. Res.* **46,** 1211–1216 (1995).
3. Schönberg, C. H. L. & Loh, W. K. W. Molecular identity of the unique symbiotic dinoflagellates found in the bioeroding demosponge Cliona orientalis. *Mar. Ecol. Prog. Ser.* **299,** 157–166 (2005).
4. Wilkinson, C. R. Net primary productivity in coral reef sponges. *Science (80-.).* **219,** 410–412 (1983).
5. Pineda, M. C., Duckworth, A. & Webster, N. Appearance matters: sedimentation effects on different sponge morphologies. *J. Mar. Biol. Assoc. United Kingdom* **96,** 481–492 (2016).

Figure S1. Percentage of growth (mean ± SE) (based on sponge surface area) for all species (except for *Cliona orientalis*) and targeted treatments (SSC: 0, 3, 10, 30 and 100 mg L^{-1}) at the end of the experimental (black) and observational periods (grey).

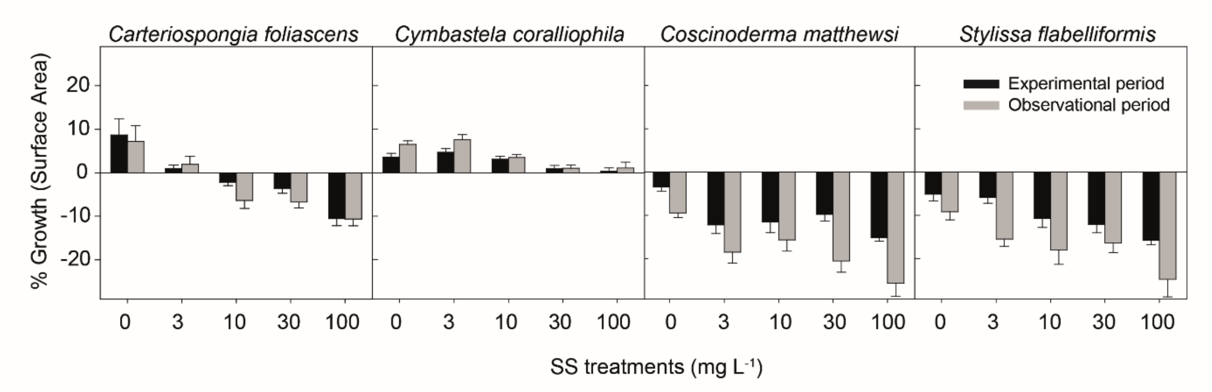

Figure S2. Percentage of mortality for each treatment across the experiment in *Carteriospongia foliascens* and *Coscinoderma matthewsi.*

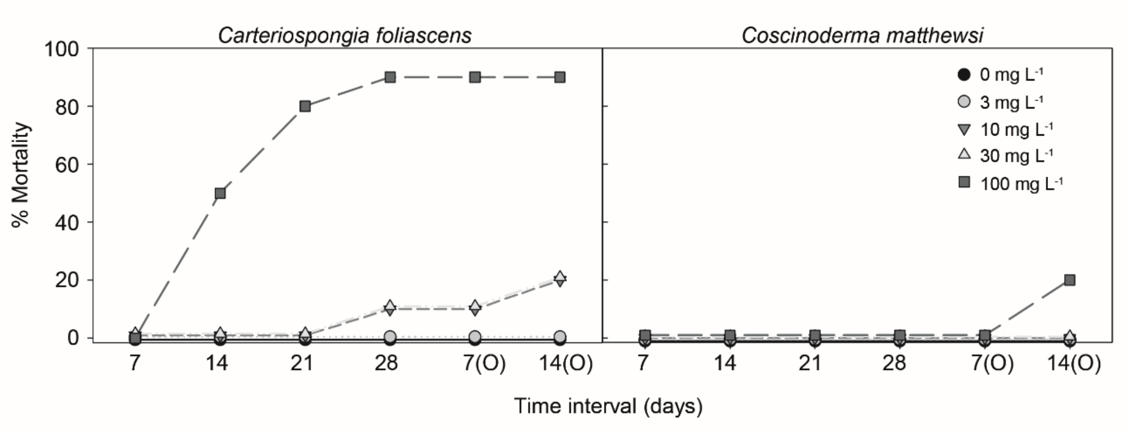

Figure S3. Histological image at 200x of *Cliona orientalis* grown in SSC of a) 0 mg L^{-1} and b) 100 mg L^{-1} for 28 days. Arrows indicate photosymbionts (pink cells) while *cc* refers to choanocyte chambers.

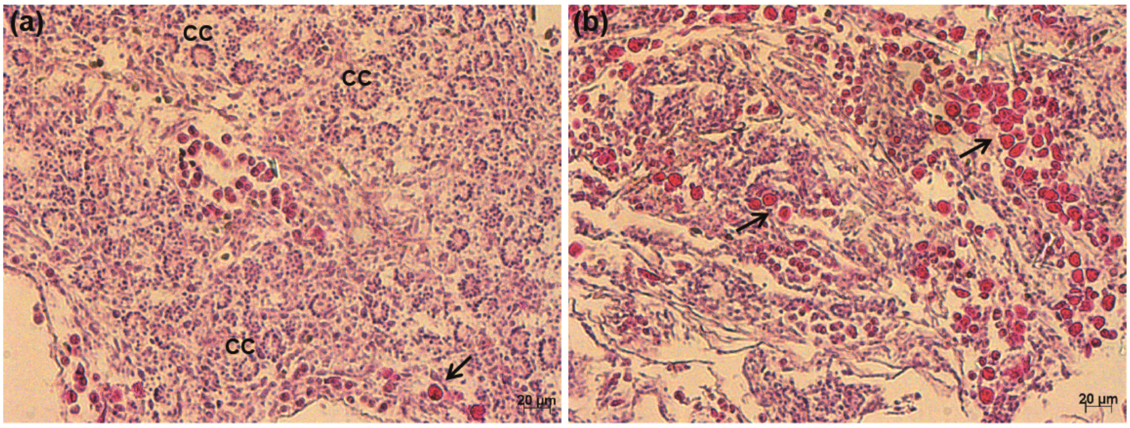

Figure S4. Mean values (± SE) of Chl d, for each phototrophic species at all SSC mg L^{-1} after the experimental and observational periods.

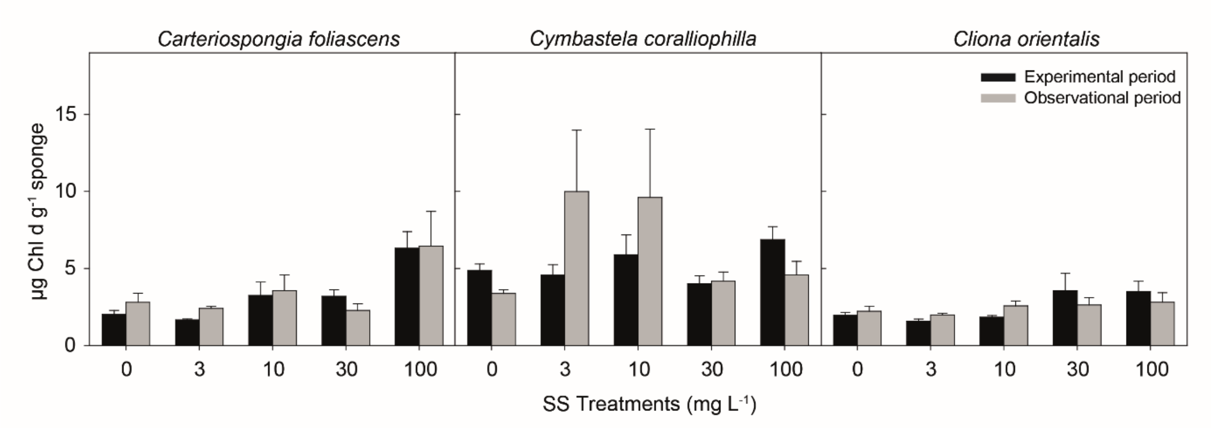

Figure S5. Non-metric Multi-Dimensional Scaling plots on microbial OTU data. nMDS of microbial communities for all 5 sponge species and the environmental control (seawater).

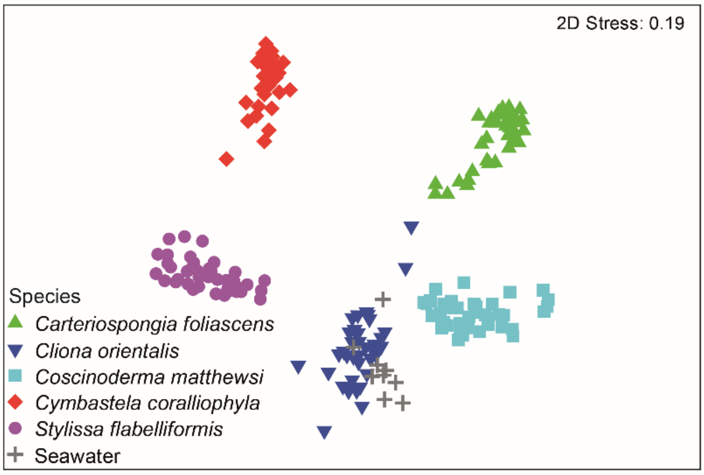

Figure S6. Phylum level bar chart for Seawater (Environmental control). Average relative abundance of each bacteria phylum (and class for Proteobacteria) for 3 replicate seawater samples within each targeted SSC (0, 30 and 100 mg L^{-1}). Only OTUs representing greater than 1% of the overall community were included.

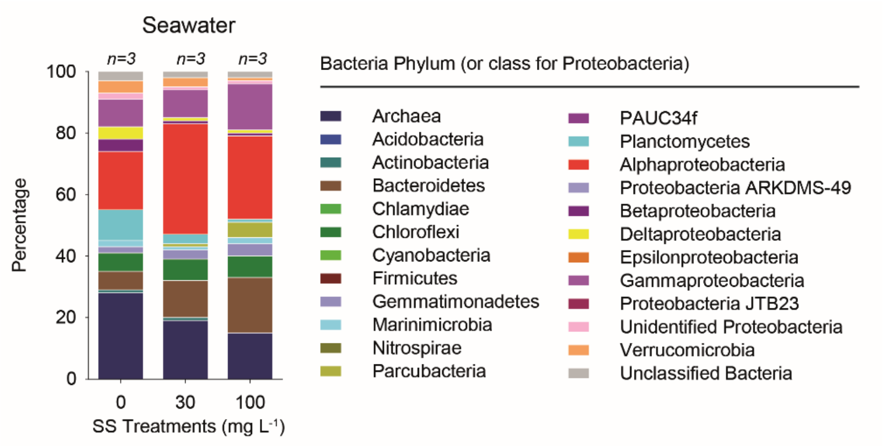

Supplement: Supplementary file 1 — Supplementary Information [file 41598_2017_5241_MOESM1_ESM.pdf]
